# Improved Dried Blood Spot PCR Assay for Universal Congenital Cytomegalovirus Screening in Newborns

**DOI:** 10.1128/spectrum.04041-22

**Published:** 2023-03-20

**Authors:** Jean H. Kim, Veronica Robles, Kristin E. D. Weimer, Lisa M. Gehtland, Katerina S. Kucera

**Affiliations:** a RTI International, Research Triangle Park, North Carolina, USA; b Duke University School of Medicine, Durham, North Carolina, USA; Quest Diagnostics

**Keywords:** congenital cytomegalovirus, cCMV, newborn screening, dried blood spots, PCR assay

## Abstract

Congenital cytomegalovirus (cCMV) is the most common perinatal infection, the leading cause of nongenetic sensorineural hearing loss, and one of the leading causes of neurodevelopmental impairment in the developed world. Early identification via newborn screening (NBS) would benefit the many undiagnosed infants who are either asymptomatic or mildly to moderately symptomatic, of whom 20% develop sequelae. The sensitivity of a recently developed PCR-based method to detect CMV in dried blood spots (DBS) is less than 80% and requires significantly more specimen than any other NBS test. We sought to improve the analytical sensitivity of the screening method by using droplet digital PCR and direct PCR and decreasing the amount of specimen utilized. The methods were tested with CMV-spiked filters, DBS from CMV-spiked cord blood, and DBS from neonates with cCMV. The results showed that the analytical sensitivity of all modified methods was equivalent to that of the reference method, with consistent CMV detection at high viral loads and inconsistent detection at low viral loads.

**IMPORTANCE** Implementation of screening for cCMV in public health programs is hindered by feasibility challenges, including limited specimen availability and an insufficiently sensitive DBS-based screening assay. We report on efforts to improve the currently available DBS-based molecular assay to increase its feasibility of implementation in newborn screening programs. Although the analytical sensitivity of the modified methods was similar at the lower IU, equivalent CMV detection was achieved using one punch instead of the required three punches for the reference method. This reduction in sample size has the potential to substantially improve feasibility of NBS for cCMV. A population-based study is needed to further evaluate the clinical sensitivity of the improved assay.

## INTRODUCTION

Congenital cytomegalovirus (cCMV) is the most common congenital viral infection worldwide, affecting 0.5% to 2.3% of births, and is the most common cause of acquired sensorineural hearing loss (1–4). Most infants with cCMV (85% to 90%) are asymptomatic at birth; however, up to 20% will have long-term sequelae, including seizures, chorioretinitis, neurodevelopmental impairment, and, most commonly, hearing loss (5, 6). Importantly, over 43% of children who are eventually diagnosed with CMV-associated hearing loss are asymptomatic at birth and are missed by the newborn hearing screen (4). Early identification can significantly improve the outcomes of infants infected with cCMV by identifying infants who could benefit from pharmacologic treatment and improving access to hearing aids, cochlear implants, and developmental services (1, 7–9). Because of this and the significant economic burden, cCMV is a candidate for universal newborn screening (NBS) (9, 10). However, there are significant challenges to be addressed before its inclusion.

Recently, Minnesota was the first U.S. state to mandate universal NBS for cCMV (5), and several other states (e.g., Utah and Connecticut) have mandates to perform targeted cCMV screening for all infants who fail their newborn hearing screen (11). Some individual hospitals in other states have also implemented targeted screening for cCMV in their newborn nurseries (12, 13). Although feasible and effective at identifying infants with cCMV, there is evidence that targeted screening programs detect far fewer infected children than likely would be identified through universal screening and can miss over 43% of infants with CMV-related hearing loss in infancy (4, 11, 12, 14).

The National CMV Foundation submitted a nomination to the Advisory Committee for Heritable Disorders in Newborns and Children for consideration for inclusion on the Recommended Uniform Screening Panel (RUSP) in 2019 and again in 2021 using saliva specimens; however, the nomination package did not advance to full evidence review (15, 16). The feasibility of saliva-based nucleic acid testing and lack of evidence of compatibility of such testing with existing NBS processes were identified as some of the major barriers for public health implementation (16). It was noted that a population-based prospective study was needed to demonstrate the feasibility of public health implementation.

The gold standard for CMV testing is a PCR assay performed on urine or saliva samples; however, neither specimen type is collected by NBS programs, and establishing the collection of a new specimen type would require a major change to NBS programs. Dried blood spot (DBS) specimens are currently collected for NBS worldwide; therefore, a CMV assay using DBS would be highly preferable for universal screening (14, 17, 18). In the CHIMES NBS study in 2017, a DBS CMV screening assay was used, but its sensitivity was low (42%) compared to that of assays using saliva (17). Recently, the sensitivity of the assay was improved. In 2021, Dollard et al. reported assay sensitivities from two laboratories (73.2% at University of Minnesota and 76.8% at CDC) in a prospective NBS study of 12,554 newborns. The combined assay sensitivity using DBS from both laboratories was 85.7% (6). Despite this recent advance, there remains a need to further improve the DBS CMV assay sensitivity and reduce the amount of specimen required for the screening test.

In this study, we evaluated approaches to optimizing the PCR-based CMV DBS assay (6) by utilizing droplet digital PCR (ddPCR), performing the assay directly on the DBS punch without nucleic acid extraction, and reducing the DBS specimen amount from three 3.2-mm punches to one. We hypothesized that assay optimization would improve the sensitivity or amount of sample needed for the detection of cCMV.

## RESULTS

### Detection of CMV from spiked filter punches.

**FIG 1:**
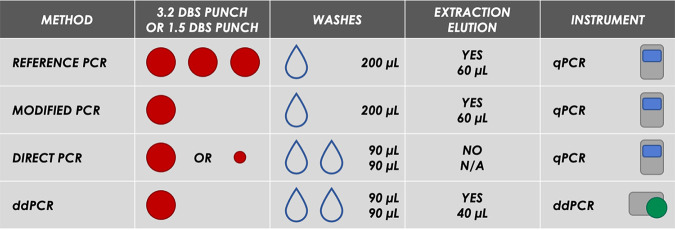
Description of the process for each test method used in the study. The large red circles represent 3.2-mm DBS punches, the small red circle represents the 1.5-mm punch, and the droplets depict the number of washes. qPCR, quantitative PCR; N/A, not applicable.

Using all four methods (modified PCR, direct PCR, ddPCR, and reference PCR) (Fig. 1), CMV was detected from the 3.2-mm punches loaded with 3.3 to 3.3 × 10^6^ copies of CMV (Table 1). Direct PCR had the highest analytical sensitivity, detecting 40% to 86% of the CMV copies loaded on the filter punches, including the second lowest loaded concentration (3.3 × 10^1^ copies/punch). The modified and reference PCR methods and the ddPCR method detected 6% to 15% and 2% to 4% of the loaded CMV copies per punch, respectively, down to the third lowest concentration (3.3 × 10^2^ copies/punch). None of the methods detected the lowest concentration (3.3 × 10^0^ copies/punch) of loaded CMV.

**TABLE 1:** CMV detection from 3.2-mm punches (without blood)

No. of CMV copies/punch	*C_T_* value for:			Detection of CMV using ddPCR
	Reference PCR^*a*^	Modified PCR^*b*^	Direct PCR	
3.3 × 10^6^	22.49	21.10	19.01	Yes
3.3 × 10^5^	25.86	24.71	23.15	Yes
3.3 × 10^4^	29.76	29.21	26.26	Yes
3.3 × 10^3^	32.90	32.90	29.61	Yes
3.3 × 10^2^	36.81	37.14	34.11	Yes
3.3 × 10^1^			37.92	No
3.3 × 10^0^				No

a Reference PCR, 3 punches.

b Modified PCR, 1 punch.

### Detection of CMV from spiked cord blood.

We applied these same four methods to detect CMV in contrived DBS using cord blood specimens spiked with CMV at concentrations of 10^2^ to 10^7^ copies per DBS. A single 3.2-mm punch was taken from each blood spot at each CMV-loaded concentration, and the results of the three test methods were compared to those from the reference PCR method, which used three punches at each CMV-loaded concentration.

**FIG 2:**
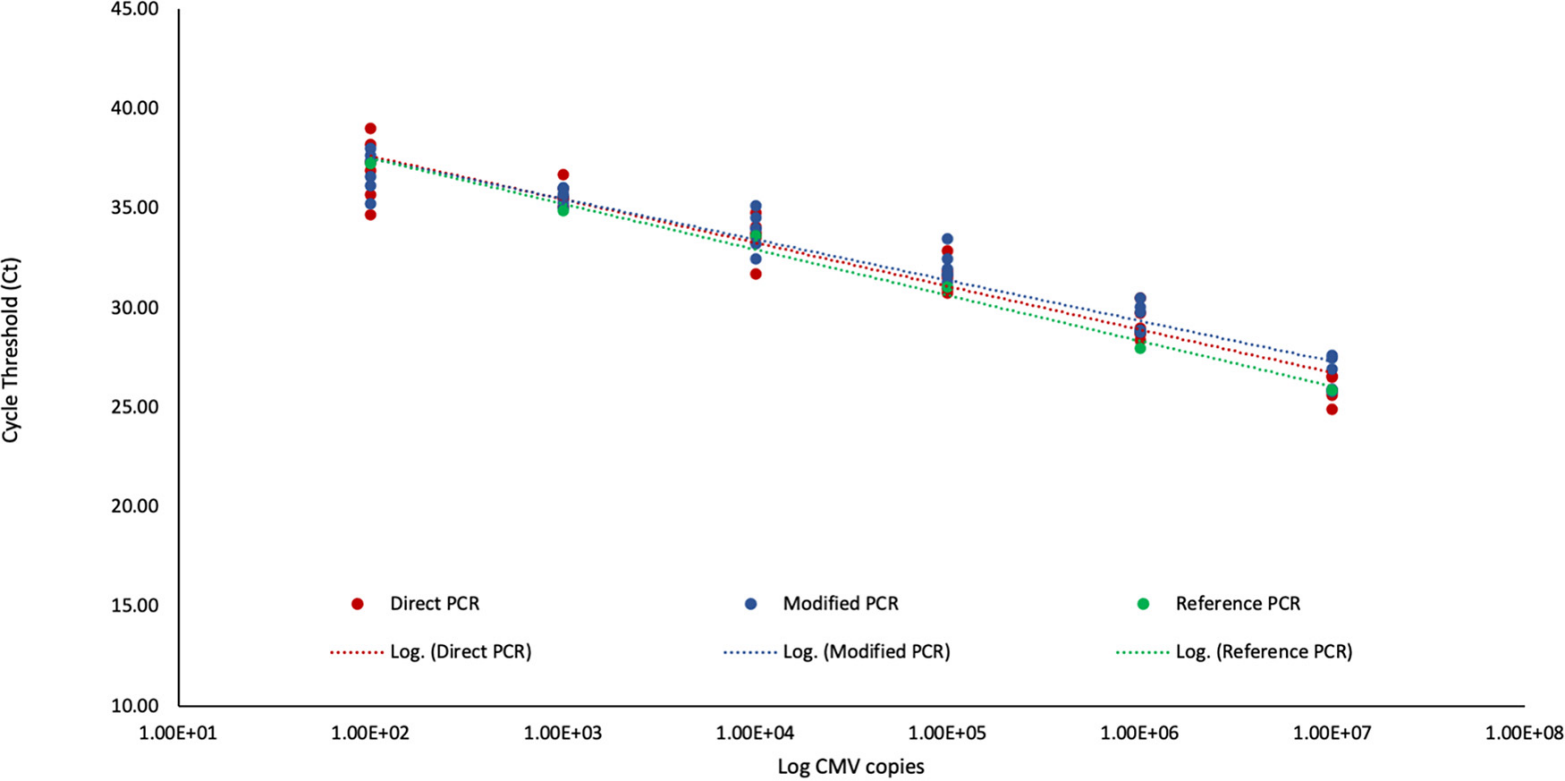
Comparison of *C_T_* values of the modified PCR, direct PCR, and reference PCR methods for detecting CMV from lab-generated spiked cord blood spots. The modified PCR and direct PCR methods had similar *C_T_* values at each of the loaded CMV copies compared to the reference PCR method.

All methods provided consistent detection of CMV from punches loaded with 10^4^ to 10^7^ copies per DBS (Table 2). The modified and direct PCR methods detected CMV at all concentrations tested, but the ddPCR method did not consistently detect CMV at the lower concentrations (10^2^ and 10^3^ copies per DBS), with no amplification in two of the six runs. Additionally, the threshold cycle (*C_T_*) values at all concentrations of CMV for the modified and direct PCR methods were similar to those of the reference PCR method (Fig. 2).

**TABLE 2:** CMV detection from lab-generated spiked cord blood spots using a single 1.5-mm or 3.2-mm punch

No. of CMV copies per DBS	No. of positive results/total no. of tests for:			
	3.2-mm punch			1.5-mm punch
	Modified PCR	Direct PCR	ddPCR	Direct PCR
10^7^	6/6	6/6	6/6	12/12
10^6^	6/6	6/6	6/6	12/12
10^5^	6/6	6/6	6/6	11/12
10^4^	6/6	6/6	6/6	11/12
10^3^	6/6	6/6	4/6	8/12
10^2^	6/6	6/6	4/6	4/12

For the direct PCR method, there was occasional interference with the fluorescence detection during amplification because of the physical presence of the punch in the reaction well, which resulted in aberrant amplification curves. We reduced the size of the punch from 3.2 mm to 1.5 mm using contrived DBS cord blood spiked with known concentrations of CMV. The smaller punches did not impede the instrument’s ability to detect fluorescence; however, the sensitivity for the detection of CMV was reduced, as the smaller punch size resulted in lower viral concentrations in the PCR (Table 2).

### Detection of CMV from clinical specimens.

A preliminary assessment of the methods in patient clinical specimens was performed using DBS generated from 10 deidentified whole blood samples from infants with cCMV, all at different stages of antiviral treatment. The viral load for the samples ranged from clinically negative (<90 IU/mL) to 2.3 × 10^5^ IU/mL (Table 3). Most of the samples were classified as detectable but not quantifiable (90 to 137 IU/mL) by routine clinical testing.

**TABLE 3:** CMV detection from patient dried blood spots using 1.5-mm and 3.2-mm punches

DBS sample ID	CMV^*a*^ (IU/mL)	CMV (IU/DBS)	No. of positive results/total no. of tests for:				
			Reference PCR^*b*^	Modified PCR^*c*^	Direct PCR		ddPCR
					3.2-mm punch	1.5-mm punch	
D1	245	12	3/9	2/9	0/3	0/3	1/3
D2	90–137	5–7	1/9	0/9	0/3	0/3	1/3
D3	Below detectable limits	Below detectable limits	0/9	2/9	0/3	1/3	1/3
D4	90–137	5–7	3/9	1/9	0/3	0/3	1/3
D5	~10^3^	~10^2^	9/9	9/9	3/3	4/4	3/3
D6	233,411	11,671	9/9	9/9	3/3	3/4	3/3
D7	90–137	5–7	0/9	0/9	0/3	0/3	0/3
D8	90–137	5–7	1/9	0/9	0/3	0/3	1/3
D9	90–137	5–7	0/9	0/9	0/3	0/3	1/3
D10	3,939	197	6/6	6/6	1/1	2/2	2/2

a Determined at the Duke Clinical Laboratory. Analytical measurement range, 137 to 9.1 million (2.1 to 7.0 log_10_) IU/mL. Test was performed using the Roche COBAS AmpliPrep/COBAS TaqMan CMV assay; 50 μL of whole blood was loaded on DBS for testing.

b Reference PCR, 3 punches.

c Modified PCR, 1 punch.

A single filter punch (a 3.2-mm punch for all methods and a 1.5-mm punch for the direct PCR method only) taken from the blood spot samples was used to assess the three test methods (modified PCR, direct PCR, and ddPCR), and the results were compared to those of the reference PCR method. All methods consistently detected CMV from either a single (modified PCR, direct PCR, and ddPCR) or a triple (reference PCR) 3.2-mm DBS punch made from blood with viral concentrations of 10^3^ to 10^5^ IU/mL (10^2^ to 10^4^ IU/DBS) (Table 3). For the smaller filter punch (1.5 mm), detection was also nearly 100% successful at these high concentrations. However, detection of CMV from blood spots with 245 IU/mL (12 IU/DBS) and below was not consistent. The modified and reference PCR methods resulted in amplification from zero to three of nine replicates, and the ddPCR method detected CMV in one of three replicates. Both the modified PCR and ddPCR methods yielded false-positive results for the clinically negative sample. The direct PCR method using the 3.2-mm and 1.5-mm punches resulted in no detection of CMV from the blood spots with 245 IU/mL and below.

### Impact of extraction modification on CMV detection.

**FIG 3:**
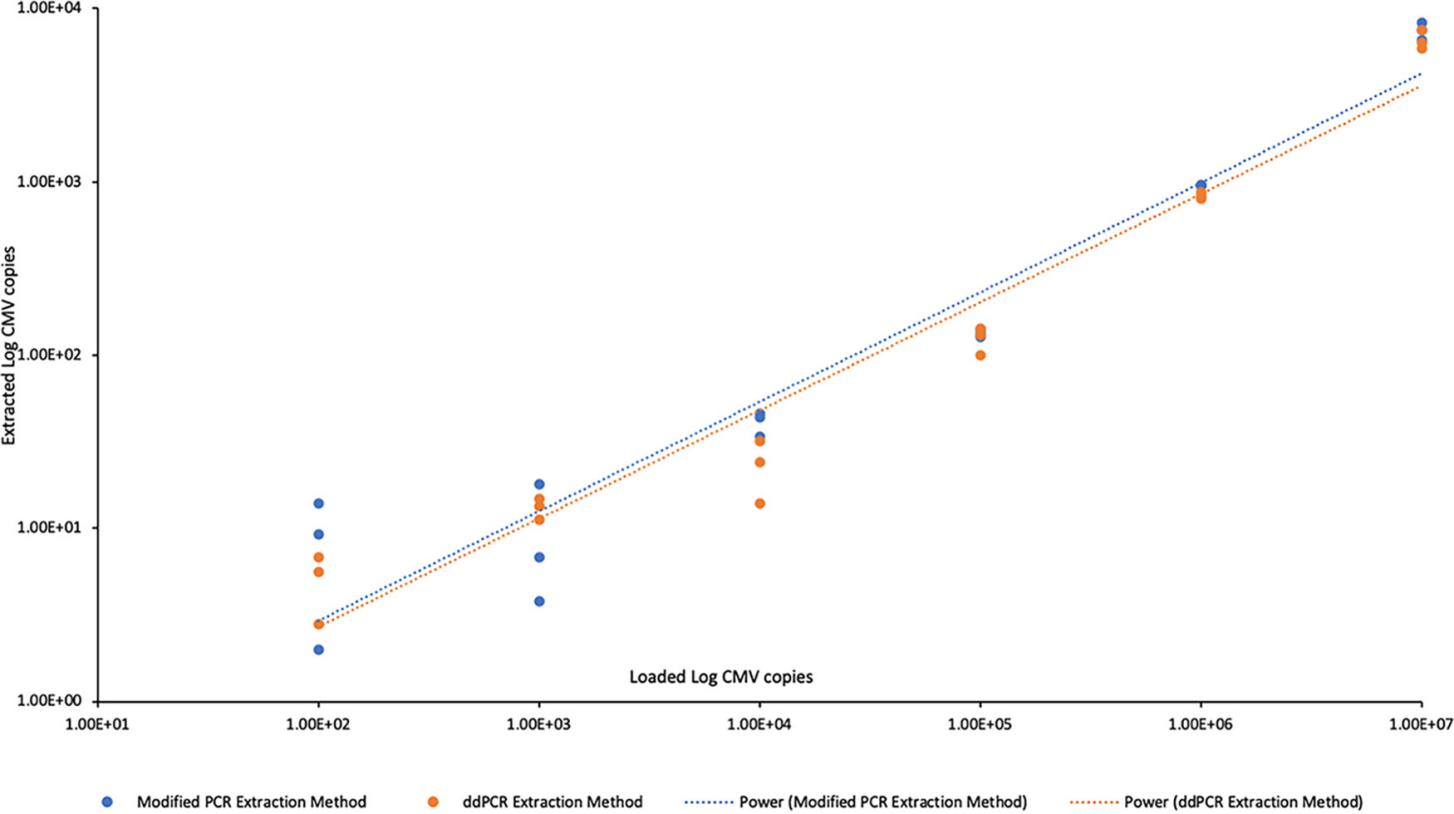
ddPCR method performance with two extraction methods (modified PCR and ddPCR extraction) using spiked cord blood spots. Both extraction methods provided similar detection of CMV.

The extraction protocol used for the ddPCR method was slightly different from that used for the modified and reference PCR methods (Fig. 1) and was designed to provide more CMV template to the ddPCR assay by reducing the volumes of the wash buffer and the final elution. We compared the two extraction protocols to see whether the procedure for the ddPCR method improved detection. Lab-generated spiked cord blood spots were used, and the extracts were assessed for CMV detection using the ddPCR assay. Extracted products from both methods yielded the same results, with very little recovery of CMV at the lower loading concentrations and with high variability (Fig. 3). These results indicate that the modifications made to the extraction protocol did not improve CMV detection with the ddPCR method compared to the protocol for the modified and reference PCR methods.

## DISCUSSION

In this study, we evaluated potential improvements to the PCR-based assay developed for NBS for cCMV by Dollard et al. (6) and compared three method variations (modified PCR, direct PCR, and ddPCR) to the reference assay (6). All three methods detected CMV at high viral loads in the presence and absence of whole blood and were equivalent to the reference PCR method. However, at low to very low viral loads in the clinical specimens, the detection of CMV was variable or undetectable by all methods, including the reference PCR method. The results for the cCMV samples and the contrived cord blood samples were not converted to IU per filter punch or DBS. Although we were not able to perform the experiment to determine the conversion factor, the multiplier is typically less than a log, which would not change the overall conclusions of this study.

Initial experiments using 3.2-mm punches spiked with known concentrations of CMV and no influence of blood suggested that the direct PCR method was more analytically sensitive for CMV detection at lower concentrations compared to the other methods. However, with the addition of whole blood, the analytical sensitivity of the direct PCR method was similar or lower, demonstrating the negative impact of the complex blood matrix, or possibly the inefficient release of CMV DNA from the punch for efficient PCR amplification to occur. Additionally, we saw interference of fluorescence detection by the 3.2-mm punch, which physically impeded the instrument’s ability to accurately measure fluorescence during amplification. The smaller punch (1.5 mm) eliminated the interference for fluorescence detection but reduced the template availability and decreased the assay sensitivity. Dissolution or homogenization of the filter paper matrix could be considered to eliminate the detection interference using the direct PCR method.

There is increasing demand on DBS quantity, given that new conditions are periodically added to NBS panels, and pilot studies are conducted to support future additions. One 3.2-mm DBS punch for the initial screening test for all current RUSP conditions is standard, three 3.2-mm punches if retested in duplicate, including multiplex tests designed to detect multiple conditions from a single punch. The CDC cCMV assay that we aimed to improve in this study requires three 3.2-mm punches for the initial test, nine 3.2-mm punches if retested in duplicate. The modified PCR method using a single 3.2-mm punch performed similarly to the reference PCR method that requires three 3.2-mm punches, suggesting that the sample volume can be reduced to a single punch without compromising analytical sensitivity, a potential significant advance in implementation feasibility. However, a larger study is needed to further evaluate the clinical sensitivity when using three DBS punches versus one.

ddPCR is a unique assay that partitions a single sample into thousands of reaction droplets individually assessed for PCR-positive or -negative amplification. The design for the assay suggests a level of sensitivity exceeding that of quantitative PCR (qPCR), especially with lower template availability—in this case, lower viral concentrations. However, in this study, the sensitivity for CMV detection was not improved at any viral concentration compared to the other methods, and false positives were observed.

One factor that plays an important role in the sensitivity of PCR-based methods is DNA extraction (19). We tested several modifications to the extraction protocol used in the reference method (Fig. 1), including longer incubation times (data not shown); however, none of the changes improved the detection of CMV. Substantial variability of CMV detection was observed for all methods, especially at low viral loads, which is likely a combination of the limitations of the tested DNA extraction methods and the uneven distribution of viral particles across each DBS.

We found that DBS loaded with high CMV viral loads were consistently detectable using all methods (modified PCR, direct PCR, ddPCR, and reference PCR), but detection of the low viral loads was variable. The direct PCR method, although initially promising, did not detect low viral loads of CMV in the presence of whole blood, and the ddPCR method did not perform as expected, despite the assay’s unique methodology, which was designed to increase analytical sensitivity. Although we were unable to increase the analytical sensitivity at the lower IU values, we were able to demonstrate equivalent CMV detection using a single punch instead of the three punches. This reduction in sample size substantially improves the feasibility of NBS for cCMV with DBS specimens.

## MATERIALS AND METHODS

We performed three methods and compared the results to the reference standard screening assay developed and published by CDC (the fourth method) (6). For the purposes of this study, the methods were designated (i) the modified PCR method, (ii) the direct PCR method, (iii) the ddPCR method, and (iv) the reference PCR method.

### CMV stock, spiked filter paper, and clinical specimens.

**(i) cCMV samples (without blood).** CMV AD169 viral stock (7.3 × 10^8^ copies/mL) was kindly provided by the Permar lab (Duke University, Durham, NC). The viral stock was 10-fold serially diluted using reagent grade water (Invitrogen, Waltham, MA) and used to spike prepunched (3.2-mm) blank filters (Whatman 903 Proteinsaver cards; Cytiva, Marlborough, MA). Each 3.2-mm filter punch was inoculated with 4.5 μL of the diluted virus. This provided 3.3 to 3.3 × 10^6^ viral copies per filter punch.

**(ii) Contrived cord blood samples.** Ten milliliters of neonatal cord blood was collected in EDTA tubes from the discarded placentas of CMV-negative infants. The cord blood was spiked with 10-fold serially diluted CMV AD169. The viral load of each spiked sample was confirmed using qPCR as previously described. Briefly, viral DNA was extracted from 400 μL of whole blood using the High Pure viral nucleic acid kit (Roche Life Science; Indianapolis, IN), and qPCR was performed using primers targeting the highly conserved immediate early 2 (IE2) exon 5 region (17). DBS were generated using 50 μL of spiked (or negative-control) cord blood applied to filter paper at concentrations of 10^2^ to 10^7^ copies per DBS (20, 21), from which 3.2-mm and 1.5-mm punches were used for testing.

**(iii) CMV-positive patient samples.** Scavenged whole blood from infants (≤1 year old) with cCMV was obtained from discarded blood collected for routine clinical testing. DBS were generated using 50 μL of blood, and punches (3.2 mm and 1.5 mm) were obtained for testing. The CMV load for each whole blood sample was determined in the clinical laboratory using the Roche COBAS AmpliPrep/COBAS TaqMan CMV assay, with an analytical measurement range of 137 to 9.1 million (2.1 to 7.0 log_10_) IU/mL, and recorded by the study team. These values were used to calculate the CMV load per DBS. Additionally, the study team recorded whether the infant was on treatment for CMV at the time of collection. Blinded DBS samples were transferred to RTI in batches for testing. The study was approved by the Duke Institutional Review Board with a waiver of consent.

### CMV extraction from punches.

DNA was extracted from three 3.2-mm punches combined in one extraction tube for the reference PCR method and one 3.2-mm punch for the modified PCR and ddPCR methods (Fig. 1). The punches were washed once for the reference and modified PCR methods and twice for the ddPCR method. For the single wash, 200 μL of Extracta DBS buffer (Quantabio, Beverly, MA) was added, and for the double washes, 90 μL of the buffer was added each time. The punches with buffer were centrifuged at 2,250 × *g* for 5 min, and the eluents were discarded. For the extraction, 60 μL of Extracta was added to the washed DBS for the reference and modified PCR methods, and 40 μL was added for the ddPCR method. The punches were briefly centrifuged to collect the liquid and placed on a thermocycler at 95°C for 25 min. The extracts were transferred to clean plates and either used immediately or stored at −20°C.

### CMV detection by PCR and ddPCR.

All methods used the same primers and probe for the detection of CMV (6, 22). The primers (200 nM) and probe (900 nM) targeted the viral IE2 gene: IE2 forward, GAG CCC GAC TTT ACC ATC CA; IE2 reverse, CAG CCG GCG GTA TCG A, and probe, VIC-ACC GCA ACA AGA TT-MGBNFQ.

For the direct PCR method, one filter punch (1.5 or 3.2 mm) was placed in a well of a 96-well plate and washed twice with 90 μL Extracta DBS buffer with shaking at 2,500 rpm, initially for 10 min and then for 5 min, discarding the eluent after each shake. The washed punches were left in the wells, and 15 μL (1.5-mm punch) or 40 μL (3.2-mm punch) of PCR master mix (PerfeCTa qPCR Toughmix, uracil-DNA glycosylase [UNG], low ROX, with primers and probe; Quantabio) was added directly to the wells. The plate was incubated on the QuantStudio 7 Pro system (Thermo Fisher, Waltham, MA) using the following protocol: 45°C for 2 min, 95°C for 20 min, and 45 cycles of 95°C for 15 ss and 60°C for 1 min.

For the reference and modified PCR methods, 10 μL of the extracted product, described above, was mixed with 30 μL of PCR master mix (PerfeCTa qPCR Toughmix with primers and probe). The plate was incubated on the QuantStudio 7 Pro system using the following protocol: 50°C for 2 min, 95°C for 10 min, and 45 cycles of 95°C for 15 s and 60°C for 1 min.

For the ddPCR method, 10 μL of the extracted product, described above, was added to 12 μL of ddPCR master mix (ddPCR Supermix [Bio-Rad, Hercules, MA] with primers and probe). The mixture was vortexed for 10 s and briefly centrifuged. The combined product was added to a droplet cartridge with 70 μL of droplet oil and loaded onto the droplet generator (Bio-Rad). The droplets were gently transferred to a ddPCR plate, heat sealed with foil, and placed into the deep-well C1000 thermocycler (Bio-Rad), using the following protocol: 90°C for 1 min, 40 cycles of 94°C for 30 s and 60°C for 1 min, and 98°C for 10 min. The plate was then placed on the QX200 ddPCR system (Bio-Rad) to be counted.
